# Trends in the Use, Sociodemographic Correlates, and Undertreatment of Prescription Medications for Chronic Obstructive Pulmonary Disease among Adults with Chronic Obstructive Pulmonary Disease in the United States from 1999 to 2010

**DOI:** 10.1371/journal.pone.0095305

**Published:** 2014-04-21

**Authors:** Earl S. Ford, David M. Mannino, Anne G. Wheaton, Letitia Presley-Cantrell, Yong Liu, Wayne H. Giles, Janet B. Croft

**Affiliations:** 1 Division of Adult and Community Health, National Center for Chronic Disease Prevention and Health Promotion, Centers for Disease Control and Prevention, Atlanta, Georgia, United States of America; 2 Department of Preventive Medicine and Environmental Health, University of Kentucky College of Public Health, Lexington, Kentucky, United States of America; Pulmonary Research Institute at LungClinic Grosshansdorf, Germany

## Abstract

**Background:**

The extent to which patients with COPD are receiving indicated treatment with medications to improve lung function and recent trends in the use of these medications is not well documented in the United States. The objective of this study was to examine trends in prescription medications for COPD among adults in the United States from 1999 to 2010.

**Methods:**

We performed a trend analysis using data from up to 1426 participants aged ≥20 years with self-reported COPD from six national surveys (National Health and Nutrition Examination Survey 1999–2010).

**Results:**

During 2009–2010, the age-adjusted percentage of participants who used any kind of medication was 44.2%. Also during 2009–2010, the most commonly used medications were short-acting agents (36.0%), inhaled corticosteroids (ICS) (18.3%), and LABAs (16.7%). The use of long-acting beta-2 agonists (LABAs) (p for trend <0.001), ICS (p for trend = 0.013) increased significantly over the 12-year period. Furthermore, the use of tiotropium increased rapidly during this period (p for trend <0.001). For the years 2005–2010, the use of LABAs, ICS and tiotropium increased with age. Compared with whites, Mexican Americans were less likely to use short-acting agents, LABAs, ICS, tiotropium, and any kind of COPD medication. Among participants aged 20–79 years with spirometry measurements during 2007–2010, the use of any medication was reported by 19.0% of those with a moderate/severe obstructive impairment and by 72.6% of those with self-reported COPD and any obstructive impairment.

**Conclusion:**

The percentages of adults with COPD who reported having various classes of prescription medications that improve airflow limitations changed markedly from 1999–2000 to 2009–2010. However, many adults with COPD did not report having recommended prescription medications.

## Introduction

Because of the chronic and often progressive nature of COPD, treatment is paramount in reducing symptoms, improving quality of life and health status, enhancing exercise capacity, slowing deterioration of lung function, limiting exacerbations, and prolonging the life of patients with COPD [1], [2]. Management of these patients encompasses several critical elements which include 1) reducing or preferably eliminating exposure to the offending agent that caused COPD; 2) prescribing medications as indicated; 3) use of oxygen when indicated; 4) pulmonary rehabilitation; and 5) reducing exacerbations. Treatment options for prescription medications directed at the underlying airflow limitation have evolved over time; whereas the mainstay of treatment several decades ago included primarily methylxanthines and short-acting beta-2 agonists, treatment has gradually shifted to emphasize increasingly the use of long-acting beta-2 agonists and inhaled corticosteroids (ICS).

As the therapeutic armamentarium of medications used to treat COPD has expanded, guidelines concerning the use of these agents have also changed [1], [3]. Consequently, a current perspective on the use of medications used to treat COPD in the United States is valuable in gauging the concordance between recommended guidelines and indicators of clinical practice. Therefore, the principal objective of this study was to examine the trend in the usage of prescription medications used to treat the airflow limitation in patient with COPD in national samples of adults with COPD. As secondary objectives, we sought to examine sociodemographic determinants of the usage of these medications and to determine the percentage of participants with an airflow limitation who were receiving treatment with medications for their condition.

## Methods

### Ethics Statement

The present study used publically-available data and was exempt from human subjects review.

The analyses in this study were conducted using six 2-year cycles of data from the National Health and Nutrition Examination Survey (NHANES) NHANES 1999–2010. In each of these cycles, a stratified multistage selection process was used to select a national probability sample. Participants who agreed to participate were interviewed in their homes and offered an opportunity to attend an examination in the mobile examination center. Those who agreed were asked to complete additional questionnaires, to undergo various examinations, and to provide a blood sample in the mobile examination center. Response rates for the interview ranged from 78% to 84%, and response rates for the examination ranged from 75% to 80%. Details about the surveys may be found elsewhere [4], [5].

A participant was defined with self-reported COPD if the survey participant reported having been told that he or she had chronic bronchitis and still had it or if he or she reported that he or she had emphysema. Participants who reported not having been told that they had either condition were defined as not having COPD.

During 2007–2010, participants in NHANES aged 6–79 years were offered an opportunity to have spirometry. Participants were asked to provide three acceptable maneuvers on Ohio 822/827 dry-rolling seal volume spirometers [6]. Detailed protocols for the spirometric examination can be found elsewhere [6], [7]. Only results from quality spirometric efforts were used.

Equations derived from earlier NHANES III data (1988–1994) were used to calculate the predicted forced expiratory volume in 1 second (FEV1) and forced vital capacity (FVC) [8]. We applied the Global Initiative for Chronic Obstructive Lung Disease (GOLD) classification of COPD severity that is based on post-bronchodilator spirometric results to pre-bronchodilator testing [2]. In addition, we also defined a category of restrictive impairment (FEV1/FVC ≥0.70 and FVC <80% predicted) for some analyses. In addition, some analyses were conducted using FEV1 predicted of <60% and 60–<80% in accordance with the 2011 American College of Physicians, American College of Chest Physicians, American Thoracic Society, and European Respiratory Society (ACP/ACCP/ATS/ERS) recommendations [1].

Participants who responded affirmatively were then asked to report the medications that they were using and, when available, to show the medication containers to the interviewer who could record up to 20 medications. The following major groups of medications were established: any short acting medication (albuterol, fenolterol, levalbuterol, metaprotenerol, pirbuterol, terbutaline, ipratropium, fenolterol plus ipratropium, and albuterol plus ipratropium), long-acting beta-2 agonists (LABA) (salmeterol, formoterol, and arformoterol whether used separately or as part of a combination product), ICS (beclomethasone, budenoside, flunisolide, fluticasone, and triamcinolone whether used separately or as part of a combination product), short-acting anticholinergic agents (ipratropium), long-acting anticholinergic agents (tiotropium), methylxanthines (aminophylline and theophylline), and any of the aforementioned medications.

Participants who responded affirmatively to one or more of these questions were considered to have evidence of respiratory symptoms. Because the question about shortness of breath was asked only of participants aged ≥40 years, analyses involving respiratory symptoms were limited to this age group.

To examine the sociodemographic variation in prescribed medication use, we included the following covariates: age, gender, self-reported race or ethnicity (white, African American, Mexican American, and other), and educational level (less than 12 years, high school graduate or equivalent, education beyond high school).

The analyses were limited to participants aged ≥20 years for analyses involving adults with self-reported COPD in 1999–2010, to participants aged 20–79 years in 2007–2010 for analyses involving spirometric data, and to participants aged 40–79 years for analyses involving respiratory symptoms. Age-adjustment to the projected year 2000 U.S. population was done with the direct method using three age groups (20–39 years, 40–59 years, and ≥60 years). Tests for linear trend were conducted using polynomial orthogonal contrasts. In addition, we examined tests for linear trend by calculating prevalence ratios adjusted for age, gender, race or ethnicity, and educational status using log-binomial models. To examine the most recent sociodemographic correlates of medication use, we combined data for the years 2005–2010 to increase sample size. We used SAS for data processing and SUDAAN to account for the complex sampling design of the survey and produce estimates (means and percentages) that are representative of the civilian noninstitutionalized population in the United States. Sample sizes provided in the text and tables represent unweighted numbers.

## Results

The numbers of participants aged ≥20 years who were interviewed for the six consecutive cycles were 4880, 5411, 5041, 4979, 5935, and 6218, and the numbers of participants who reported having been told that they had COPD were 213 (weighted percentage 4.2%), 208 (3.5%), 234 (4.3%), 212 (4.4%), 314 (4.3%), and 245 (3.3%). Medication status could be ascertained for all but two participants. The sociodemographic breakdown for these participants with COPD is shown in **Table 1**. Based on aggregated data for the 12-year period, adults with self-reported COPD were older, more likely to be women, to be white, to be less educated, and to be a current smoker than adults without COPD (Table 2).

**Table 1:** Unadjusted percentages (standard error) of selected characteristics of adults aged ≥20 years with self-reported chronic obstructive pulmonary disease, National Health and Nutrition Examination Survey 1999–2010.

	1999–2000	2001–2002	2003–2004	2005–2006	2007–2008	2009–2010	P Chi-square
N	212	208	234	211	314	245	
Age (years)							<0.001
20–39	21.2 (4.4)	22.7 (4.6)	14.1 (2.8)	19.8 (2.8)	9.6 (1.5)	18.1 (2.3)	
40–59	33.1 (6.5)	33.5 (4.0)	46.6 (4.6)	41.1 (2.8)	37.5 (3.2)	35.3 (2.4)	
60+	45.8 (5.3)	43.8 (5.2)	39.4 (3.5)	39.2 (3.4)	52.9 (2.7)	46.6 (2.4)	
Gender							0.446
Men	32.2 (3.6)	36.3 (5.0)	39.2 (3.7)	40.5 (4.3)	42.5 (3.9)	34.0 (4.1)	
Women	67.8 (3.6)	63.7 (5.0)	60.8 (3.7)	59.5 (4.3)	57.5 (3.9)	66.0 (4.1)	
Race or ethnicity							0.635
White	79.2 (3.6)	77.9 (5.1)	83.6 (3.9)	80.1 (2.9)	81.4 (4.7)	77.5 (3.5)	
African American	6.9 (2.3)	8.1 (2.5)	10.3 (3.0)	9.3 (2.4)	9.8 (2.4)	13.4 (2.5)	
Mexican American	2.9 (0.9)	1.6 (0.4)	2.5 (0.9)	1.8 (0.6)	1.9 (0.8)	1.9 (1.0)	
Other	11.0 (3.8)	12.4 (5.9)	3.6 (1.7)	8.7 (2.2)	6.9 (2.5)	7.2 (1.3)	
Education							<0.001
<High school	40.4 (2.9)	27.0 (4.3)	31.2 (6.0)	19.6 (2.2)	39.9 (4.0)	34.9 (3.9)	
High school graduate	30.3 (3.1)	32.2 (4.1)	21.4 (3.4)	32.4 (3.4)	25.3 (3.1)	27.3 (4.9)	
>High school	29.3 (3.8)	40.8 (4.8)	47.4 (5.6)	48.0 (2.8)	34.8 (4.3)	37.7 (4.2)	
Smoking status							0.054
Current	43.0 (4.4)	37.2 (4.6)	33.2 (5.7)	49.2 (4.3)	36.4 (3.8)	47.4 (4.0)	
Former	32.9 (4.3)	30.8 (4.3)	36.3 (4.5)	29.4 (3.9)	41.8 (3.7)	32.6 (3.7)	
Never	24.0 (2.2)	32.1 (3.8)	30.5 (5.0)	21.4 (4.0)	21.7 (2.4)	20.1 (2.8)	

*Sample sizes represent unweighted numbers.

† Sample size for all six surveys is 1423.

**Table 2:** Selected characteristics, by self-reported chronic obstructive pulmonary disease status, National Health and Nutrition Examination Survey 1999–2010.

	Chronic obstructive pulmonary disease (%, SE)	No chronic obstructive pulmonary disease (%, SE)	P-value
Age (years)			
20–39	17.2 (1.3)	40.3 (0.5)	<0.001
40–59	38.1 (1.7)	37.9 (0.4)	0.964
60+	44.7 (1.6)	21.8 (0.5)	<0.001
Gender			
Men	37.7 (1.7)	48.4 (0.2)	<0.001
Women	62.3 (1.7)	51.6 (0.2)	<0.001
Race or ethnicity			
White	80.1 (1.7)	69.9 (1.3)	<0.001
African American	9.6 (1.0)	11.3 (0.7)	0.431
Mexican American	2.1 (0.3)	8.0 (0.6)	<0.001
Other	8.1 (1.3)	10.8 (0.8)	0.011
Education			
<High school	32.2 (1.8)	19.3 (0.5)	<0.001
High school graduate	28.0 (1.5)	25.1 (0.5)	0.570
>High school	39.8 (1.8)	55.6 (0.8)	<0.001
Smoking status			
Current	41.0 (1.8)	23.0 (0.5)	<0.001
Former	34.2 (1.6)	24.1 (0.5)	<0.001
Never	24.8 (1.4)	52.9 (0.6)	<0.001

Number of adults with COPD varied from 1424 to 1426. Number of participants without COPD varied from 30947 to 31034.

### Medication Use among Participants with Self-reported COPD: NHANES 1999 to 2010

During 2009–2010, an unadjusted 53.2% (SE 4.9) of participants reported no medications used in the management of COPD, 21.1% (SE 3.4) reported a medication from one group, 16.9% (SE 2.9) reported medications from 2 groups, and 8.7% (SE 3.3) reported medications from 3 or more groups. Averaged over all 12 years, 7.6% (SE 0.9) of participants used medications from 3 or more groups.

During 2009–2010, an age-adjusted 44.2% of participants used any type of prescription medication used to treat COPD (**Table 3**). In descending order of reported use were short-acting agents (36.0%) consisting primarily short-acting beta-2 agonists (SABA) (31.3%), ICS whether alone or in combination with a LABA (18.3%), LABAs whether alone or in combination with an inhaled corticosteroid (16.7%), tiotropium (10.5%), and methylxanthines (<2.0%). The trend in the use of any medication increased significantly during the study period. The relatively high use of medications during 1999–2000 was a function of the high use of short-acting agents. Significant increases were noted for the use of LABAs, ICS, tiotropium, and any medication. The data also suggested that the use of ICS as single agents decreased whereas the use of combination products of LABAs and corticosteroids increased (data not shown).

**Table 3:** Age-adjusted percentages (standard error) of adults aged ≥20 years with self-reported chronic obstructive pulmonary disease taking prescription medications for chronic obstructive pulmonary disease, National Health and Nutrition Examination Survey 1999–2010.

	1999–2000	2001–2002	2003–2004	2005–2006	2007–2008	2009–2010	P for linear trend†
N*	212	208	234	211	314	245	
Any short-acting agent‡	31.9 (4.7)	19.6 (3.1)	26.0 (4.7)	26.6 (5.1)	33.0 (4.0)	36.0 (5.9)	0.076
Any inhaled long-acting beta-2 agonists§	–**	9.1 (2.2)	11.1 (2.4)	8.4 (3.2)**	15.5 (2.1)	16.7 (2.7)	<0.001
Any inhaled corticosteroid?	11.8 (2.0)	12.8 (2.1)	13.6 (2.4)	9.6 (3.6)**	21.3 (3.8)	18.3 (2.3)	0.013
Tiotropium	0.0	0.0	–††	–††	4.9 (1.1)	10.5 (2.4)	<0.001
Methylxanthines¶	–††	–††	3.1 (0.5)	–††	–††	1.7 (0.5)	0.016
Any medication for COPD	35.9 (4.6)	21.7 (3.2)	29.0 (4.8)	29.6 (5.6)	37.5 (4.7)	44.2 (5.9)	0.028

*Sample sizes represent unweighted numbers.

† From log-binomial analysis with adjustment for age, gender, and race or ethnicity.

‡ Albuterol, fenolterol, levalbuterol, metaprotenerol, pirbuterol, terbutaline, ipratropium.

§ Salmetrol, formoterol, arformoterol.

? Beclomethasone, budenoside, flunisolide, fluticasone, triamcinolone.

¶ Aminophylline, theophylline.

**Relative standard error ≥30% to <40%.

†† Relative standard error ≥40%.

Even after combining six years of data, the numbers of participants in the 2005–2010 time period who reported using various types of medications was limited resulting in reduced power to examine sociodemographic correlates of medication use (**Table 4**). The use of ICS and tiotropium increased among successive age groups in 2005–2010. Compared with whites, Mexican Americans were less likely to use short-acting agents, LABAs, ICS, tiotropium, and any kind of COPD medication (**Table 4**).

**Table 4:** Adjusted prevalence ratios* (95% confidence interval) for use of prescription medications used to treat chronic obstructive pulmonary disease among 770 adults aged ≥20 years with self-reported chronic obstructive pulmonary disease, National Health and Nutrition Examination Survey 2005–2010.

	Any short-actingagent†	Any inhaled long-actingbeta-2 agonists‡	Any inhaledcorticosteroid§	Tiotropium	Any medication
Number using medication	253	129	153	65	318
Age (years)					
20–39	1.00	1.00	1.00	1.00	1.00
40–59	1.01 (0.70, 1.48)	1.26 (0.61, 2.61)	1.43 (0.76, 2.68)	7.49 (1.45, 38.70)	1.08 (0.76, 1.52)
60+	1.01 (0.70, 1.47)	1.78 (0.98, 3.23)	1.92 (1.10, 3.34)	9.26 (2.08, 41.10)	1.24 (0.87, 1.77)
Gender					
Men	1.03 (0.77, 1.37)	0.89 (0.59, 1.37)	0.88 (0.58, 1.34)	0.95 (0.48, 1.87)	1.06 (0.82, 1.37)
Women	1.00	1.00	1.00	1.00	1.00
Race or ethnicity					
White	1.00	1.00	1.00	1.00	1.00
African American	1.16 (0.88, 1.54)	1.02 (0.63, 1.67)	1.04 (0.63, 1.71)	0.81 (0.30, 2.22)	1.08 (0.82, 1.42)
Mexican American	0.37 (0.15, 0.89)	0.33 (0.12, 0.90)	0.33 (0.15, 0.70)	0.14 (0.02, 0.97)	0.36 (0.18, 0.72)
Other	1.24 (0.88, 1.76)	1.48 (0.84, 2.61)	1.28 (0.79, 2.06)	0.81 (0.33, 2.00)	1.08 (0.78, 1.49)
Education					
<High school	1.19 (0.87, 1.61)	1.36 (0.85, 2.19)	1.30 (0.89, 1.91)	2.17 (1.02, 4.63)	1.20 (0.91, 1.58)
High school graduate	1.16 (0.88, 1.53)	1.27 (0.70, 2.30)	0.89 (0.51, 1.53)	1.29 (0.59, 2.84)	1.08 (0.84, 1.40)
>High school	1.00	1.00	1.00	1.00	1.00

*Prevalence ratios are adjusted for all variables shown in table.

† Albuterol, fenolterol, levalbuterol, metaprotenerol, pirbuterol, terbutaline, ipratropium.

‡ Salmetrol, formoterol, arformoterol.

§ Beclomethasone, budenoside, flunisolide, fluticasone, triamcinolone.

After combining 12 years of data for participants aged ≥40 years with self-reported COPD, 87.1% (SE 1.5) of participants who did not use medications to treat COPD and 95.2% (SE 1.3) of those who used such medications reported having one or more of the following symptoms: cough, phlegm, wheezing, and shortness of breath.

### Medication Use among Participants with an Obstructive Impairment: NHANES 2007 to 2010

Of the 11289 participants aged 20–79 years who were interviewed in 2007–2010, 10981 attended the mobile examination center and 9047 had values for FEV1 and FVC, allowing their lung function status to be established for 7887 participants with adequate spirometric efforts: 580 participants with a mild obstructive impairment, 428 with a moderate obstructive impairment, and 63 with a severe obstructive impairment. Because of the small number of participants with a severe obstructive impairment, they were combined with participants who had a moderate obstructive impairment.

The prevalence of self-reported COPD was 3.7% and that of any obstructive impairment was 13.4% (7.6% mild obstructive impairment, 5.8% moderate or worse obstructive impairment). Of adults with any obstructive impairment based on spirometry, 7.5% had self-reported COPD, and among adults with self-reported COPD, 34.8% had evidence of an obstructive impairment. The use of any COPD medication was reported by 7.3% (SE 2.2) of participants with a mild obstructive impairment and 19.0% (SE 2.7) of participants with a moderate/severe obstructive impairment (**Table 5**). Among those with a moderate/severe obstructive impairment, the most commonly reported medications were short-acting agents (15.5%), followed by ICS (9.0%), and LABAs (6.5%). Tiotropium and methylxanthines were used by small percentages of participants (≤2%).

**Table 5:** Age-adjusted percentages (standard error) of adults aged 20–79 years taking prescription medications for chronic obstructive pulmonary disease defined by spirometry, by Global Initiative for Chronic Obstructive Lung Disease status, National Health and Nutrition Examination Survey 2007–2010.

	Normal	Mild OI	≥Moderate/severe OI
N*	6293	580	491
Any short-acting agent†	2.2 (0.2)	–¶	15.5 (2.4)
Any inhaled long-acting beta-2 agonists‡	1.1 (0.2)	–¶	6.5 (1.5)
Any inhaled corticosteroid§	1.6 (0.2)	–¶	9.0 (2.0)
Tiotropium	–¶	–¶	–¶
Methylxanthines?	–¶	–¶	–¶
Any medication for COPD	3.1 (0.3)	7.3 (2.2)¶	19.0 (2.7)

OI = obstructive impairment.

*Sample sizes represent unweighted numbers.

† Albuterol, fenolterol, levalbuterol, metaprotenerol, pirbuterol, terbutaline, ipratropium.

‡ Salmetrol, formoterol, arformoterol.

§ Beclomethasone, budenoside, flunisolide, fluticasone, triamcinolone.

? Aminophylline, theophylline.

¶ Relative standard error ≥30%.

Over 70% of participants with an obstructive impairment and with self-reported COPD reported using any COPD medication, the majority of which were short-acting agents (**Table 6**). In comparison, almost 30% of adults without an obstructive impairment but with self-reported COPD used a medication.

**Table 6:** Age-adjusted percentages (standard error) of adults aged 20–79 years taking prescription medications for chronic obstructive pulmonary disease, by obstructive impairment and self-reported chronic obstructive pulmonary disease, National Health and Nutrition Examination Survey 2007–2010.

	OI-PFT +, COPD-SELF +	OI-PFT +, COPD-SELF −	OI-PFT −, COPD-SELF +	OI-PFT −, COPD-SELF −
N*	100	971	147	6669
Any short-acting agent†	68.1 (3.9)	8.1 (1.8)	27.4 (5.7)	1.9 (0.2)
Any inhaled long-acting beta-2 agonists‡	16.3 (8.9)	2.9 (0.8)	11.3 (3.0)	1.1 (0.2)
Any inhaled corticosteroid§	23.6 (9.7)	3.8 (1.0)	12.6 (3.0)	1.5 (0.2)
Tiotropium	–¶	–¶	–¶	–¶
Methylxanthines?	–¶	–¶	–¶	–¶
Any medication for COPD	72.6 (3.6)	9.6 (1.8)	29.1 (5.8)	2.8 (0.2)

COPD = chronic obstructive pulmonary disease; OI = obstructive impairment; PFT = pulmonary function test.

*Sample sizes represent unweighted numbers.

† Albuterol, fenolterol, levalbuterol, metaprotenerol, pirbuterol, terbutaline, ipratropium.

‡ Salmetrol, formoterol, arformoterol.

§ Beclomethasone, budenoside, flunisolide, fluticasone, triamcinolone.

? Aminophylline, theophylline.

¶ Relative standard error ≥40%.

In an attempt to assess the percentage of participants with COPD who reported using medications consistent with the 2011 ACP/ACCP/ATS/ERS recommendations, we stratified medications use by self-reported COPD status, presence or absence of respiratory symptoms, and percent FEV1 predicted (**Table 7**). Although the use of long-acting medications (31.5% among participants with an FEV1 predicted 60% to 80% and 53.8% of participants with an FEV1 predicted <60%) and any medications (46.2% among participants with an FEV1 predicted 60% to 80% and 72.8% of participants with an FEV1 predicted <60%) increased as FEV1% predicted decreased among participants with self-reported COPD and symptoms, substantial percentages of these participants did not report using such medications.

**Table 7:** Unadjusted percentages (standard error) of adults aged 40–79 years taking prescription medications for chronic obstructive pulmonary disease, by COPD status defined by spirometry, status of respiratory symptoms, and %FEV1 predicted, National Health and Nutrition Examination Survey 2007–2010.

	Long-acting agents†				Any medications			
	FEV1 predicted				FEV1 predicted			
	N*	60% to <80%	N*	<60%	N*	60% to <80%	N*	<60%
COPD								
Symptoms	53	31.5 (8.4)	45	53.8 (10.6)	53	46.2 (7.7)	45	72.8 (9.0)
No symptoms	4	–‡	2	–‡	4	–‡	2	–‡
No COPD								
Symptoms	349	9.8 (2.1)	87	13.0 (3.3)	349	15.7 (2.2)	87	22.6 (3.7)
No symptoms	297	–‡	25	–‡	297	1.7 (0.3)	25	–‡

COPD = chronic obstructive pulmonary disease; FEV1 =  forced expiratory volume in 1 second.

*Sample sizes represent unweighted numbers.

† LABAs, tiotropium, or corticosteroids.

‡ Relative standard error ≥40%.

## Discussion

Our analyses highlight several findings of interest. First, the types of medications that adults with self-reported COPD reported using changed substantially from 1999 to 2010. In particular, the use of LABAs, inhaled corticosteroids, and tiotropium increased sharply. Second, Mexican American adults with self-reported COPD were less likely to report using the different medications that we examined than white adults. Third, many adults with COPD were not receiving potentially beneficial treatment.

Although important changes in medications to treat COPD have occurred during the last decade, the trends in the use of these medications in the United States have not been adequately documented in the medical literature. The Food and Drug Administration approved several medications during the past decade including the combination of salmeterol and fluticasone in 2000, tiotropium in 2004, and the combination of formoterol and budesonide in 2006. The classes of all of these agents showed strong increases during the study period. A previous analysis of data from the National Ambulatory Medical Care Survey from 1995 to 2004 showed that prescriptions for oral corticosteroids in men and methylxanthines in men and women had decreased but prescriptions for anticholinergic agents had increased [9]. As in other studies, we found that short-acting agents, principally SABAs, were the most commonly used medications by 2009–2010 [10], [11]. In our study, the next most frequently used medications were combination products containing LABAs and a corticosteroid (13.7%) followed by ICS (8.0%).

Our analysis of sociodemographic correlates of medication use indicated that there were no statistically significant differences in the use of these medications between white and African American adults. However, Mexican American adults with COPD were less likely than whites to use major groups of medications. Because little information about health disparities involving treatment for COPD in Hispanic populations appears to have been published, our findings require confirmation. Reduced access to health care, language barriers, and cultural beliefs are possible factors that can affect treatment for COPD among Mexican Americans [12].

In our study, considerable percentages of adults with self-reported COPD or with COPD defined by spirometry did not divulge having medications used to treat COPD. Because of the cross-sectional nature of the survey, the presence of symptoms and use of medications were collected at a single point in time, precluding establishing the temporal sequence of symptom onset and instituted treatment. Other studies have also found low rates of pharmacotherapy among patients with COPD [13]–[15]. In the United States, an analysis of the PharMetrics database from 2004–2005 showed that 59.1% of commercial patients and 66.0% of Medicare patients with COPD were not prescribed any COPD medications [15]. In a Japanese study, 31% of patients with moderate to severe COPD did not receive adequate treatment (Takahashi 2003) [13]. In a study from Denmark, only 42% of patients who were identified as having COPD in a population study were receiving treatment in the year following their examination (Ingebrigtsen 2013) [14].

Raising awareness of COPD in the general population and clinical community is a theme that echoes with regularity in the medical literature and is critical to bringing patients with COPD into the medical system so that appropriate treatment of their condition can be initiated [16]–[18]. High levels of undiagnosed COPD or misdiagnosis of the disease likely account for some of the suboptimal rates of treatment among people with COPD [19]. In our study, we observed that a high percentage of adults whose spirometric examination indicated an obstructive impairment did not report having COPD. Although in a survey of British general practitioners, levels of confidence in their ability to diagnose and manage COPD were quite high [20], estimates suggest that about half of patients with COPD may not be diagnosed [21], [22].

Once diagnosed, other factors may influence the rate of medication use by people with diagnosed COPD shown in this and other studies. First, many people with COPD may not have received treatment as recommended by prevailing guidelines because physicians may be unaware or unfamiliar with guidelines or because some may not follow the guidelines [23], [24]. Although the reasons why physicians may not follow prevailing guidelines are not entirely clear, many aspects of guidelines were developed on the basis of key clinical trials that often excluded participants with comorbidities or other characteristics relevant to daily clinical practice [25]. A literature review of potential barriers to physician adherence to clinical practice guidelines in general identified several barriers: familiarity, agreement, self-efficacy, outcome expectancy, ability to overcome inertia of previous practice, and absence of external barriers to perform recommendations [26]. When presented with several COPD case studies, however, many practitioners tended to gravitate towards a diagnosis of cardiac rather than pulmonary origin [20]. By increasing the detection rate of COPD, which would presumably result in increased treatment, the treatment rate among all people with COPD would be increased. Other studies specific to adherence to COPD guidelines noted that agreement with recommendations, low self-efficacy, perceived outcome expectancy, resource availability, and time constraints were barriers to physician adherence to guidelines for COPD [27], [28].

Empowering patients with COPD may boost the percentage of patients who are receiving appropriate treatment for COPD and contribute to improving the course of their disease. Public health strategies to empower patients with COPD [29] complemented by clinical strategies founded in the chronic care model that seek to enhance patient self-management may serve to increase the percentages of adults with COPD on appropriate treatment [30].

Adherence to prescribed medications is suboptimal among patients with COPD [15]. Patient-associated factors that may impede adherence to prescribed treatments may include cost, complexity of treatment regime, presence of comorbidities requiring treatment, side effects, adverse events, treatment fatigue, and other innate factors. Understanding the issues is a prerequisite to developing sound interventions to maximize adherence.

In the PharMetrics database, adherence varied from 52.3% for short-acting anticholinergic agents to 82.2% for leukotriene modifiers among commercial patients and from 51.8% for short-acting anticholinergic agents to 85.2% for leukotriene modifiers among Medicare patients [15]. An analysis of another administrative database in the United States showed that adherence as measured by the proportion of days covered was highest for medications that are supposed to be used once per day compared to medications that are to be taken twice, three times, and four times per day [31]. Furthermore, adherence declined steadily from 3 months post initiation to 12 months post initiation in each category of frequency of use.

Our results are subject to several limitations. First, the small number of survey participants with COPD limited our ability to stratify the data or to examine specific medications in most instances. Second, a number of participants had a reversible airflow limitation because we used prebronchodilator spirometry results. Third, some proportion of patients with self-reported COPD do not meet spirometric criteria for obstructed airflow. For the years 2007–2010, 34.8% of participants with self-reported COPD had an obstructive impairment, and thus, the level of undertreatment of people with self-reported COPD may not be as substantial as it appears. If the proportion of adults with self-reported COPD who did not have an obstructive impairment based on spirometry remained reasonably constant over time, however, the trends in medication usage likely reflect the direction of actual trends in the population. Fourth, we did not have access to data that would have allowed us to establish the reasons why certain medications were prescribed or the route of administration. Knowing the reason for use is especially critical for medications with a wide range of uses such as oral corticosteroids.

In conclusion, substantial changes in the use of medications to treat airflow limitations among adults with COPD occurred from 1999–2000 to 2009–2010, and a low percentage of adults with self-reported COPD reported having medications used to treat COPD. Given the demonstrated benefits of treatment, improving treatment is a high public health priority as envisioned in the Public Health Strategic Framework for COPD Prevention [32]. Furthermore, these findings provide important information to guide national education efforts such as the National Heart, Lung, and Blood Institute’s COPD: Learn More, Breathe Better campaign [33]. Such efforts could enhance the ability of health professionals to increase the delivery of optimal treatment of persons diagnosed with COPD to optimum treatment.
